# The NMDA Receptor Antagonist Memantine Modulates Aging and Stress Resilience

**DOI:** 10.1111/acel.70303

**Published:** 2025-11-28

**Authors:** Vaida Juozaityte, Chiara Pregnolato, Steffen Abay‐Nørgaard, Dylan Matthew Rausch, Rebecca L. McIntyre, Zachary Gerhart‐Hines, Tune H. Pers, Anna Elisabetta Salcini, Christoffer Clemmensen

**Affiliations:** ^1^ Novo Nordisk Foundation Center for Basic Metabolic Research, Faculty of Health and Medical Sciences University of Copenhagen Copenhagen Denmark; ^2^ Biotech Research and Innovation Center (BRIC), Faculty of Health and Medical Sciences University of Copenhagen Copenhagen Denmark

## Abstract

Aging is associated with a progressive decline in physiological resilience, often linked to impaired stress responses and metabolic dysfunction. In *Caenorhabditis elegans* ( *C. elegans* ), caloric restriction (CR) and pharmacological interventions are widely used to dissect conserved longevity pathways. Here, we identify the N‐methyl‐D‐aspartate receptor (NMDAR) antagonist memantine as a novel modulator of lifespan and stress tolerance in *C. elegans* . Memantine, but not ketamine, extends median lifespan and reproductive lifespan, suggesting that the observed effects are not shared with ketamine at the tested concentration. Transcriptomic analysis revealed significant overlap between memantine‐treated animals and CR models, particularly *eat‐2* mutants, implicating shared metabolic and longevity‐associated pathways. Functionally, memantine was found to reduce mitochondrial and oxidative stress, while enhancing β‐oxidation of fatty acids, and modifying behavioral responses to food cues, delaying food‐seeking behavior and increasing locomotion under starvation, without affecting lipid storage. In summary, these findings suggest that memantine promotes stress resilience and healthy aging via metabolic changes that overlap with CR‐associated pathways, highlighting its potential as a longevity‐modulating intervention.

## Graphical abstract

Memantine, an NMDA receptor antagonist, extends lifespan and enhances stress resilience in *C. elegans* through caloric restriction–like metabolic adaptations, including reduced oxidative stress and increased fatty acid β‐oxidation, revealing a conserved mechanism linking glutamatergic signaling to healthy aging.

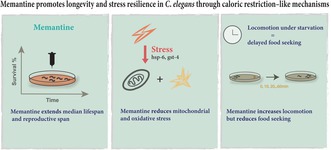

Aging is a complex biological phenomenon characterized by the gradual decline of physiological functions. Due to its short lifespan and well‐characterized genetic background, *C. elegans* has emerged as a key model organism for dissecting mechanisms of aging, including how organisms manage and tolerate cellular stress throughout life. Cellular stress response pathways, such as the heat shock response, insulin/IGF‐1 signaling, and mitochondrial stress responses, are highly conserved in *C. elegans* , making it a valuable tool for studying how these pathways influence aging across species (Park et al. 2017).

Caloric restriction (CR), a classic and widely recognized intervention for extending lifespan and enhancing healthspan across various species, has been extensively studied in *C. elegans* (Mörck and Pilon 2007; Calabrese et al. 2024). In this model organism, mutations in the *eat‐2* gene, which encodes subunits of a nicotinic acetylcholine receptor, lead to reduced pharyngeal pumping rate and decreased food intake (Avery 1993; McKay et al. 2004). These *eat‐2* loss‐of‐function worms, widely used as a genetic model of reduced food intake and dietary‐restriction‐associated lifespan extension, consistently demonstrate extended lifespan and enhanced resistance to age‐related physiological decline, highlighting the positive impact of dietary restriction on longevity and health (Lakowski and Hekimi 1998).

In parallel, pharmacological interventions targeting key signaling pathways—such as rapamycin for mTOR inhibition, metformin for AMPK activation, NAD+ precursors for mitochondrial support, and senolytics for clearing senescent cells—have emerged as promising strategies for promoting longevity and mitigating age‐associated functional decline (Mishra et al. 2022). However, while preclinical studies show encouraging results, robust clinical evidence confirming their effectiveness in humans remains limited. Given these uncertainties, there is growing interest in exploring alternative pathways that may influence aging and resilience. Among these, N‐methyl‐D‐aspartate receptor (NMDAR) antagonists, such as memantine and ketamine, have drawn attention for their neuroprotective, stress‐modulatory, and health‐promoting effects (Lipton 2006; Li et al. 2010; Lv et al. 2023). These compounds are clinically used to treat conditions such as neurodegenerative diseases and depression, owing to their ability to regulate excitotoxicity, synaptic plasticity, and neuroinflammation (Robinson and Keating 2006; Kim et al. 2019).

Beyond their established therapeutic roles, recent evidence suggests that NMDAR antagonists may exert broader effects on health, influencing cellular stress responses, metabolic regulation, and systemic inflammation. Notably, memantine has been shown to induce weight loss in rodents (Petersen et al. 2024; Huang et al. 2021). Transcriptomic studies in mammalian systems have begun to shed light on these mechanisms, but their relevance to organismal aging, reproductive dynamics, and systemic metabolic adaptation remains largely unexplored. Leveraging the tractability of *C. elegans* allows us to extend these insights by uncovering conserved, whole‐organism pathways that may not be apparent in rodent or human datasets.

Here, we demonstrate that memantine, but not ketamine, extends median lifespan and alters reproductive dynamics in *C. elegans* , suggesting a compound‐specific mechanism of action. Importantly, RNA‐sequencing analysis revealed that memantine‐treated worms exhibit gene expression patterns overlapping with those of CR‐associated models, such as *eat‐2* mutants, pointing to potential metabolic and longevity‐associated pathways influenced by NMDAR modulation. Finally, memantine reduces mitochondrial and oxidative stress while leaving endoplasmic reticulum (ER) stress unchanged. Additionally, memantine‐treated worms exhibit altered behavioral and physiological responses to food availability, including delayed food‐seeking behavior and increased locomotion under starvation conditions. Despite these behavioral changes, lipid storage remains unaffected, whereas fatty acid β‐oxidation is upregulated, suggesting metabolic remodeling that supports energy demands. Together, these findings indicate that memantine modulates aging and stress resilience via mechanisms that converge on CR‐associated pathways, complementing existing mammalian transcriptomic studies and providing new insights into its potential as a longevity‐promoting intervention.

## 1 Results

### 1.1 Memantine Extends Lifespan and Alters Reproductive Dynamics in *C. elegans*

**FIGURE 1:**
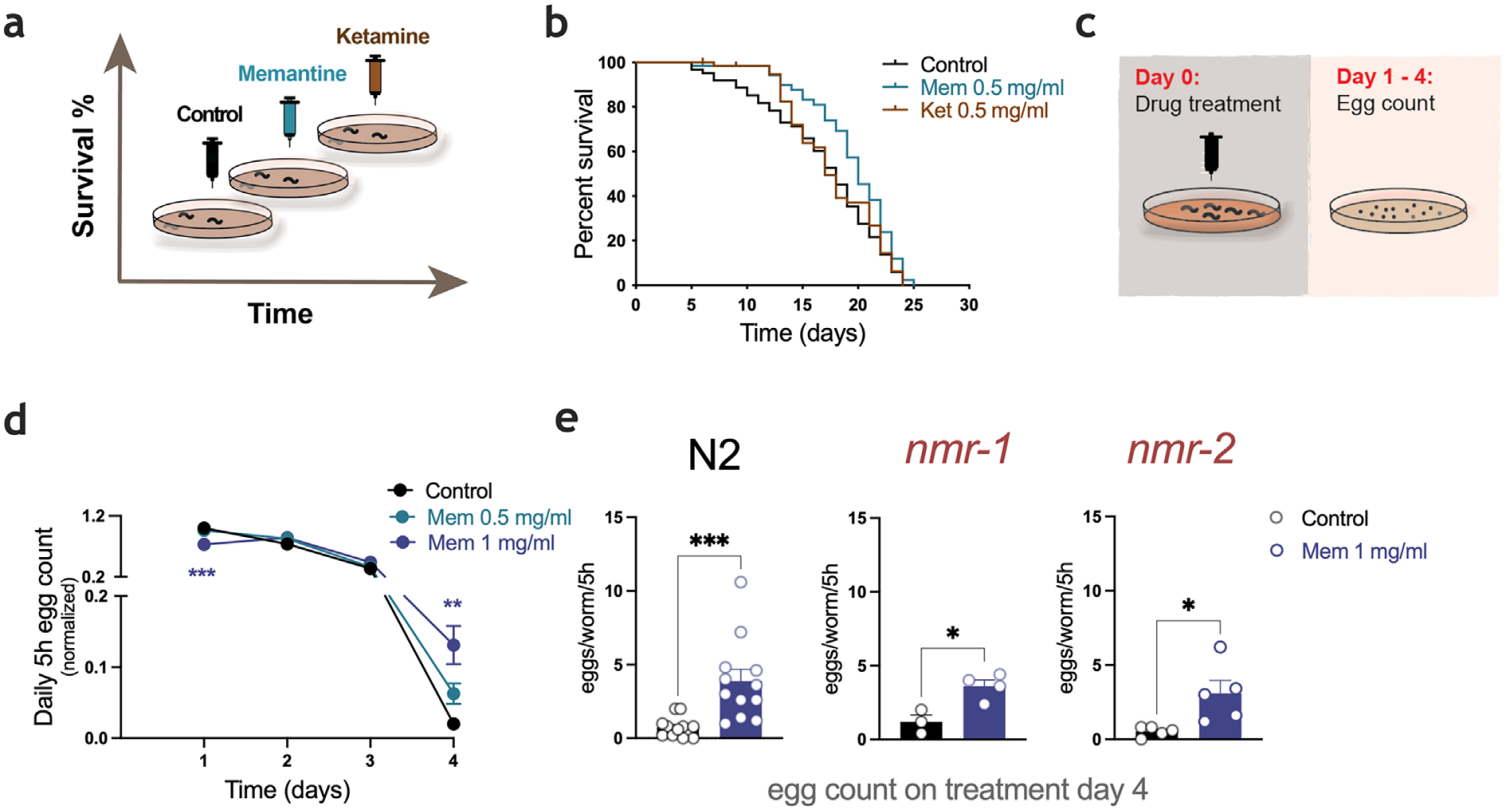
Lifespan and reproductive lifespan characterization after memantine exposure. (a) Schematics of a lifespan assay (b) Lifespan assay. Adult worms (L4 + 1 day) were treated with memantine (0.5 mg/mL), ketamine (0.5 mg/mL) or control (saline). Treatment with memantine results in median lifespan extension (*n* = 63/control; *n* = 62/memantine group, *n* = 62/ketamine group, *p < 0.05). Survival analysis was conducted using the Kaplan–Meier method, and statistical significance was analyzed by a log‐rank (Mantel‐Cox) test. (c–e) Progeny size characterization, (c) Schematics of progeny size assay, (d) Daily 5 h egg laying count evaluation following 1, 2, 3, and 4 days of treatment. Data analyzed using ordinary one‐way ANOVA (*n* = 140/control group, *n* = 135/mem 0.5 mg/mL group, *n* = 140/mem 1 mg/mL group **p < 0.01, ***p < 0.001). (e) 5 h egg laying count assessment after 4 days of memantine treatment in N2 and in *nmr‐1* and *nmr‐2* mutants. Data are mean ± SEM and analyzed with unpaired two‐tailed student's *t*‐test (*n* = 60/control N2 group, *n* = 60/mem 1 mg/mL N2 group, *n* = 30/control *nmr‐1* group, *n* = 40/mem 1 mg/mL *nmr‐1* group, *n* = 50/control *nmr‐2* group, *n* = 50/mem 1 mg/mL *nmr‐2* group), *p < 0.05, ***p < 0.001.

To establish the appropriate working concentration of memantine for aging assays, we first performed pilot lifespan experiments at 0.1 and 0.25 mg/mL (Figure S1a). While neither dose produced a clear increase in lifespan, a slight tendency toward lifespan extension was observed at 0.25 mg/mL. Therefore, we selected 0.5 mg/mL for subsequent experiments, as the well‐tolerated dose showing a trend toward efficacy. For comparison, ketamine was included as a control compound at the same concentration (0.5 mg/mL), given its related mechanism of action. Adult worms were treated with memantine (0.5 mg/mL), ketamine (0.5 mg/mL), or control solution (saline) from the first day of adulthood throughout their lifespan (Figure 1a,b). At the tested concentration of 0.5 mg/mL, memantine treatment significantly extended median lifespan, whereas ketamine did not show an effect (Figure 1b). We also tested whether memantine could improve learning and memory in *C. elegans* , but observed no changes in cognitive performance (Figure S2a–d).

Lifespan extension is frequently linked to alterations in reproductive aging, suggesting that mechanisms underlying somatic longevity may interact with reproductive systems to mediate survival and reproduction (Mukhopadhyay and Tissenbaum 2007). To explore the potential impact on progeny size, we performed an egg‐laying assay (Figure 1c–e). Memantine‐treated worms displayed a distinct reproductive profile, characterized by a dose‐dependent extension of their fertility window (Figure 1d,e). Specifically, while control worms ceased egg‐laying by Day 4 of treatment, memantine‐exposed worms continued to lay eggs, leading to a higher total egg count starting post Day 3 of treatment (Figure 1d).

To identify genetic mediators of this effect, we first investigated whether NMDAR receptor subunit mutants, *nmr‐1* and *nmr‐2*, also displayed this extension of their reproductive window. Notably, the increased egg laying observed in wild‐type worms (N2s) was also present in *nmr‐1* and *nmr‐2* mutants (Figure 1e), suggesting that this reproductive phenotype does not require *nmr‐1* or *nmr‐2* under these assay conditions.

### 1.2 Transcriptomic Analysis Reveals Memantine's Role in Lifespan Extension and Stress Resilience Pathways

**FIGURE 2:**
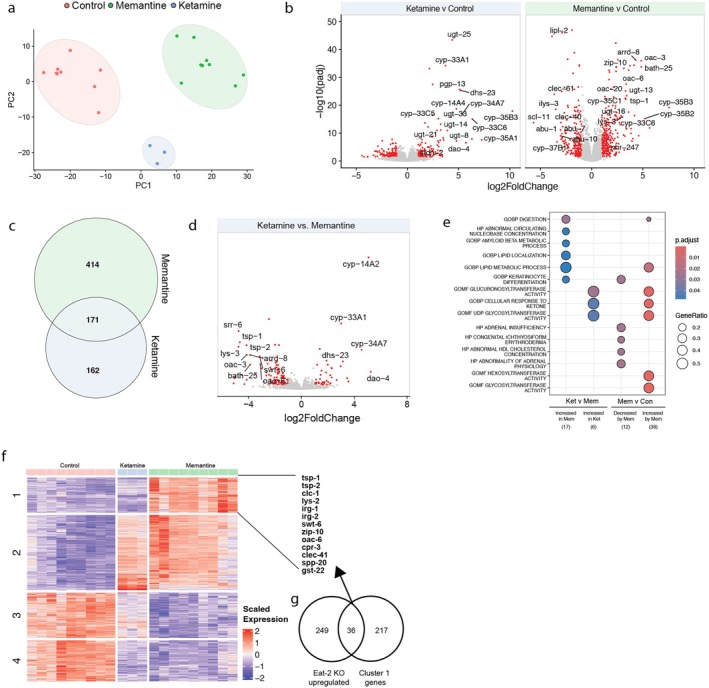
RNA sequencing analysis of memantine‐ and ketamine‐ regulated genes. (a) Principal component analysis, (b) Volcano plots, comparing ketamine versus control and memantine versus control, (c) Venn diagram of memantine versus ketamine regulated genes, (d) Volcano plot ketamine versus memantine, (e) Pathway enrichment analysis, (f) Heat map, (g) Overlap between *eat‐2* knockout upregulated (Cornwell et al. 2024) and memantine upregulated (Cluster 1 in d) genes.

Because memantine specifically extends both median lifespan and reproductive healthspan in *C. elegans* , we performed RNA‐sequencing to uncover potential molecular underpinnings, analyzing worms treated with control, memantine, or ketamine. Principal component analysis (PCA; Figure 2a) revealed distinct transcriptional responses to each compound, with memantine significantly increasing 324 transcripts and decreasing 261, compared to 164 and 169 transcripts altered by ketamine, respectively. Direct comparisons between the two treatments identified 414 genes uniquely regulated by memantine (229 lfc > 0, 185 lfc < 0) and 162 (74 lfc > 0, 88 lfc < 0) genes by ketamine (Figure 2b,c). Pathway enrichment analysis highlighted memantine‐specific activation of genes involved in lipid metabolic processes, while ketamine primarily induced glucuronosyltransferase pathways (Figure 2e). Hierarchical clustering further revealed a distinct memantine‐specific gene cluster, prominently featuring the evolutionarily conserved tetraspanin family gene *tsp‐1*, a key mediator of resilience in early‐life thermal stress (Figure 2d) (Jiang et al. 2024). Strikingly, this cluster was enriched for genes upregulated in *eat‐2* knockout worms, a well‐characterized longevity model that is acknowledged to mimic CR (Figure 2e) (Avery 1993; Lakowski and Hekimi 1998). Notably, this overlap included genes such as *zip‐2*, *ugt‐44*, and *gst‐22*, which are involved in detoxification, redox balance, and mitochondrial stress resistance (Asif et al. 2024; Hahm et al. 2020), suggesting that memantine modulates adaptive stress responses to promote longevity.

### 1.3 Memantine Reduces Mitochondrial and Oxidative Stress

**FIGURE 3:**
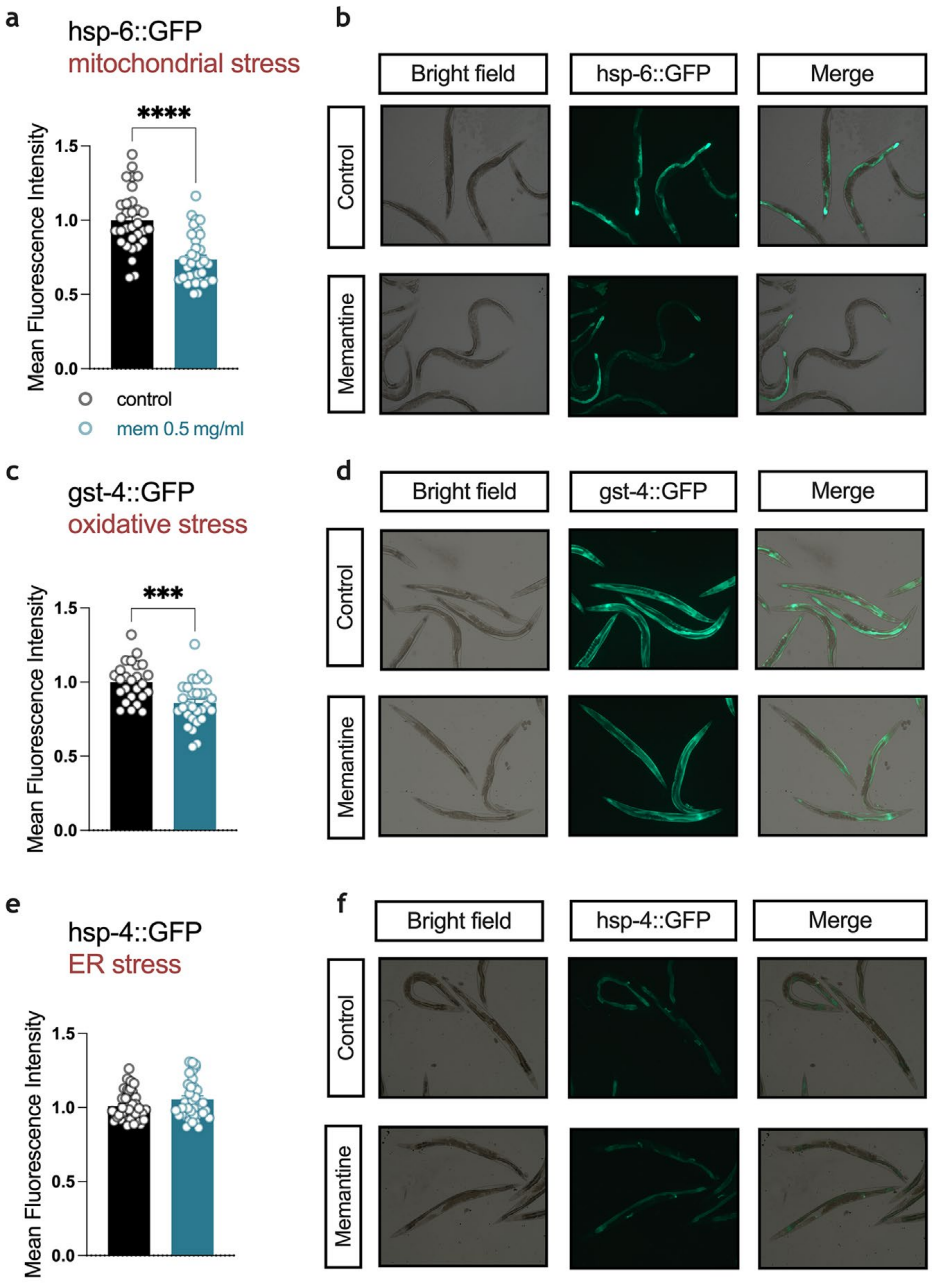
Stress response after memantine exposure. Day 1 adult worms were assessed for various stress responses following 24 h treatment with either a control (black bar) or memantine (blue bar), utilizing fluorescent stress reporter assays. (a, b) Mitochondrial stress reporter hsp‐6::GFP, *n* = 31/control group, *n* = 35/mem 0.5 mg/mL group (c) (d) Oxidative stress reporter gst‐4::GFP, *n* = 25/control group, *n* = 29/mem 0.5 mg/mL group (e, f) ER stress reporter hsp‐4::GFP *n* = 38/control group, *n* = 39/mem 0.5 mg/mL group. Data are mean ± SEM, and analyzed with unpaired two‐tailed student's *t*‐test, ***p < 0.001, ****p < 0.0001.

Building on the transcriptomic insights, we hypothesized that memantine enhances resilience to cellular stress by modulating key pathways related to mitochondrial function, oxidative stress, and ER stress. To investigate this, we assessed the impact of memantine on stress responses using fluorescent stress reporters, providing a functional evaluation of its role in cellular stress management. Mitochondrial stress, assessed by the conventional *hsp‐6*::GFP reporter (Yoneda et al. 2004), was significantly reduced in memantine‐treated worms compared to controls (Figure 3a,b). Similarly, oxidative stress levels, as measured by a well‐characterized *gst‐4*::GFP reporter (van der Hoeven et al. 2011), were decreased following memantine treatment (Figure 3c,d). To evaluate ER stress, we used the *hsp‐4*::GFP reporter (Calfon et al. 2002). In contrast to the reductions observed in mitochondrial and oxidative stress, ER stress levels remained unchanged in memantine‐treated worms (Figure 3e,f). Together, these findings suggest that memantine selectively modulates specific stress pathways including mitochondrial stress and oxidative stress.

### 1.4 Memantine Alters Behavioral and Physiological Responses to Starvation

**FIGURE 4:**
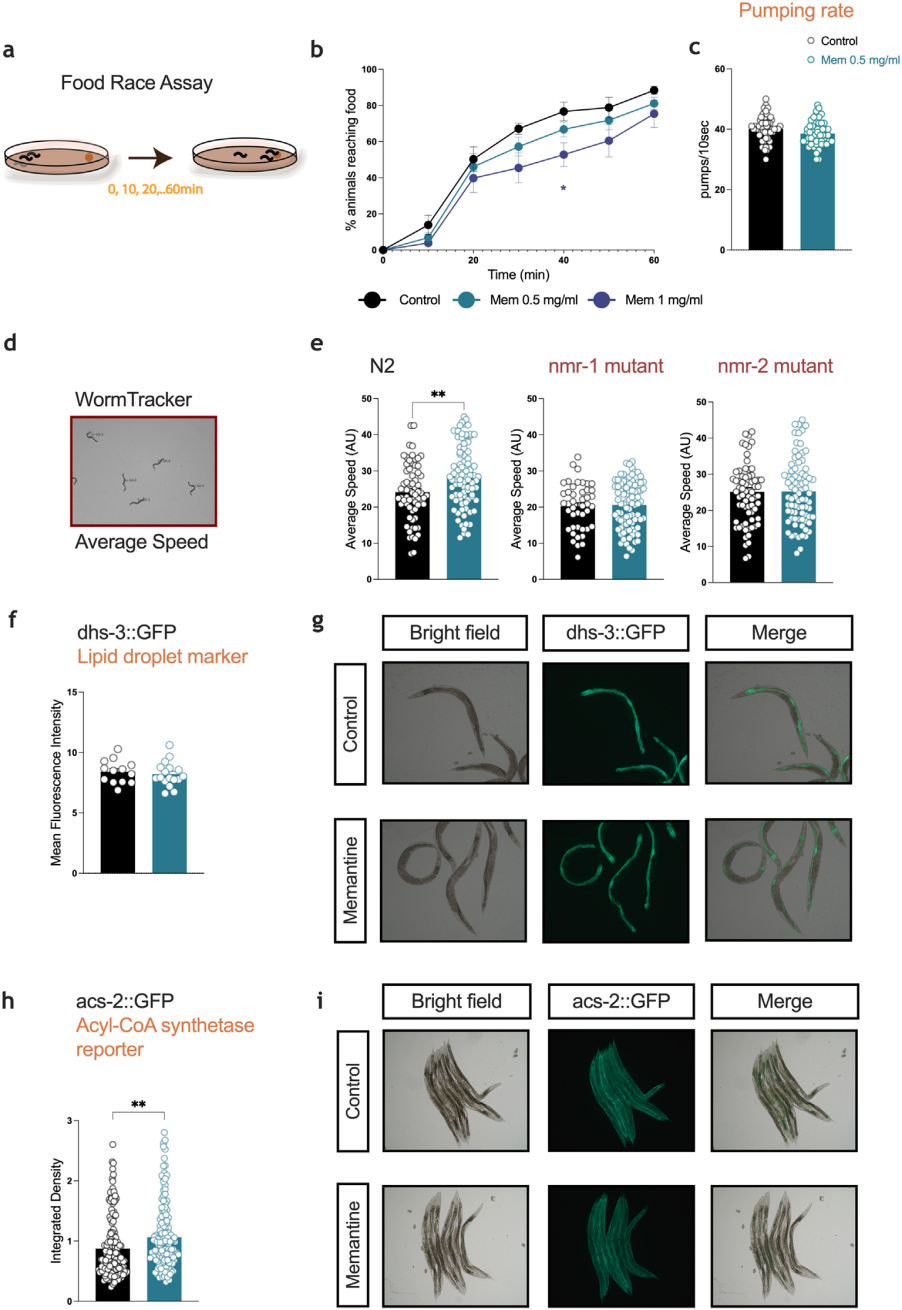
Physiological and behavioral assessment after memantine exposure. (a, b) Food race assay to evaluate the rate at which worms locate a food source following a period of starvation. (b) % of worms reaching food after exposure to memantine (0.5 mg/mL) (blue), memantine 1 mg/mL (purple) or control (black) *n* = 500/experimental group, (c) Pumping rate, measured as the number of pumps per 10‐s intervals, *n* = 55/experimental group, (d) Representative image of Worm Tracker, (e) Average speed after 1 day of treatment in wild type (N2 strain, *n* = 65/control group, *n* = 85/mem 0.5 mg/mL group), *nmr‐1* (*n* = 44/control group, *n* = 59/mem 0.5 mg/mL group) and *nmr‐2* (*n* = 70/control group, *n* = 78/mem 0.5 mg/mL group) mutants (f and g) Lipid metabolism assessment using dhs‐3::GFP reporter, *n* = 13/control group, *n* = 17/mem 0.5 mg/mL group, (f) Mean fluorescence intensity of dhs‐3::GFP, (g) Representative fluorescent images of each treatment group, (h and i) Mean fluorescence intensity of the acyl‐CoA synthetase reporter, acs‐2::GFP *n* = 148/control group, *n* = 146/mem 0.5 mg/mL group. Data in b is analyzed with 2‐way repeated measures ANOVA, *p < 0.05. Data in c, e, f and h are mean ± SEM, and analyzed with unpaired two‐tailed student's *t*‐test, **p < 0.01.

In addition to the enhanced stress response induced by memantine (Figure 3), the transcriptomic data revealed that memantine induced the expression of genes also upregulated in *eat‐2* knockout worms, a well‐characterized longevity and CR model (Figure 2g). Based on these molecular changes, we sought to investigate whether they translated into functional and behavioral adaptations. To assess the behavioral response to food availability following memantine treatment, we performed a food race assay (Figure 4a,b). Memantine‐treated worms exhibited a dose‐dependent delay in reaching the food source after a 30‐min period of starvation (Figure 4b). Despite this delay, their pharyngeal pumping rate remained unchanged, indicating that the feeding rate was unaffected (Figure 4c).

Interestingly, memantine‐treated worms demonstrated increased average locomotion speed compared to controls (Figure 4d,e). This increase in speed was abolished in *nmr‐1* and *nmr‐2* mutants, suggesting that these glutamate receptor subunits, which are functionally similar to NMDA receptors, are required for the locomotor effects of memantine (Figure 4e). Given the observed behavioral changes and the role of energy balance in locomotion and stress responses, we next evaluated lipid storage to determine whether memantine treatment altered energy reserves. Using the lipid droplet fluorescent reporter *dhs*‐*3*::GFP, we found no significant differences in lipid levels between memantine‐treated and control worms (Figure 4f,g), indicating that lipid storage was unaffected.

However, *acs*‐*2*::GFP reporter activity was increased in memantine‐treated worms (Figure 4h,i). This reporter reflects the expression of the *acs‐2* gene, which encodes an acyl‐CoA synthetase involved in fatty acid β‐oxidation, suggesting that memantine promotes enhanced metabolic remodeling to support energy demands without depleting lipid stores.

## 2 Discussion

The growing unmet need for novel targets to combat aging and age‐related diseases has driven interest in exploring unconventional compounds for their potential to enhance healthspan and extend lifespan. Here we report that memantine, a clinically approved NMDAR antagonist, significantly extends median lifespan, alters stress responses, and impacts reproductive dynamics in *C. elegans* . Notably, these effects were observed exclusively with memantine and not with ketamine, suggesting that the observed effects are not shared with ketamine at the tested concentration. The transcriptional changes and phenotypic outcomes associated with memantine treatment implicate molecular pathways commonly linked to CR, a well‐characterized intervention for promoting longevity. While transcriptomic datasets already exist for memantine in rodent models of neurodegeneration (Murakawa‐Hirachi et al. 2021; Yoo et al. 2021), these studies have not addressed organismal aging, reproductive dynamics, or systemic metabolic remodeling. Our findings therefore provide a complementary perspective, uncovering conserved CR‐associated transcriptional programs in a whole‐organism context. Together, this underscores the broader potential of memantine‐related mimetics, as a novel therapeutic strategy for age‐related diseases and longevity interventions.

Our results establish memantine as a modulator of aging, with significant extension of median lifespan in *C. elegans* . Unlike ketamine, which had no discernible impact on longevity, memantine extended both lifespan and reproductive healthspan, characterized by an extended fertility window. Interestingly, the extended egg‐laying effects persisted in NMDAR subunit mutants (*nmr‐1* and *nmr‐2*), indicating that the observed reproductive benefits are mediated via NMDAR‐independent mechanisms. This divergence may also help explain why memantine, but not ketamine, produced lifespan and resilience benefits: while both are NMDAR antagonists, memantine appears to engage additional molecular targets and pathways that ketamine does not. In rodent models, memantine is known to interact with other receptors, including serotonin 3 (5‐HT3R) and dopamine 2 (D2R) receptors, and displays distinct binding kinetics compared with ketamine (Rammes et al. 2001; Seeman et al. 2008). These properties may allow memantine to modulate systemic stress and metabolic pathways, contributing to its broader organismal effects. This decoupling of memantine's effects from its canonical role as an NMDAR antagonist highlights the importance of exploring alternative targets and pathways influenced by this compound. Future studies are needed to systematically dissect these receptor‐ and pathway‐specific mechanisms, which could reveal novel targets for promoting healthy aging. We also note that ketamine was tested only at a single concentration, so differential effects at other doses cannot be excluded.

Notably, memantine induces transcriptional changes resembling those observed in *eat‐2* mutants, a genetic model of CR (Lakowski and Hekimi 1998). In 2012, a proteomic analysis of the *eat‐2* mutant revealed a metabolic shift from carbohydrate to fatty acid metabolism, a hallmark of CR (Yuan et al. 2012). Intriguingly, memantine‐treated worms showed similar metabolic remodeling, as evidenced by pathway enrichment analysis, which highlighted enhanced lipid metabolic processes alongside adaptive stress responses. Remarkably, memantine‐treated worms also experienced a shift in reproductive lifespan similar to that observed in *eat‐2* mutants (Vora et al. 2013). Additionally, upregulated genes such as *zip‐2*, *ugt‐44*, and *gst‐22*—involved in detoxification, redox balance, and mitochondrial stress resistance—further align with the longevity‐promoting mechanisms seen in CR. These parallels suggest that memantine engages conserved transcriptional modules not previously reported in mammalian datasets, extending the scope of its known pharmacology to include systemic metabolic and aging‐related processes.

This notion was supported by functional assays demonstrating that memantine selectively modulates mitochondrial and oxidative stress pathways. Reduced *hsp‐6*::GFP and *gst‐4*::GFP fluorescence intensities in memantine‐treated worms indicate decreased mitochondrial and oxidative stress levels, respectively, while ER stress, as assessed by *hsp‐4*::GFP reporter, remained unaffected. This selective modulation underscores memantine's role in enhancing organismal resilience by targeting specific cellular stress pathways, further aligning with the hypothesis that memantine engages pathways associated with CR biology.

Memantine‐treated worms exhibited delayed food‐seeking behavior and increased locomotion speed under starvation conditions. Interestingly, the locomotor enhancement was absent in NMDAR subunit mutants, suggesting that certain behavioral effects of memantine are mediated via NMDAR‐dependent mechanisms. Despite these behavioral changes, memantine did not affect lipid storage, as assessed by *dhs‐3*::GFP fluorescence, but increased *acs‐2*::GFP reporter activity, indicating enhanced fatty acid β‐oxidation. This metabolic remodeling likely supports increased energy demands without depleting lipid reserves, aligning with the observed maintenance of energy balance and enhanced stress resilience.

In summary, our findings suggest that memantine, or novel memantine derivatives, may be promising candidates for modulating aging and healthspan through mechanisms that converge on CR‐associated pathways. The overlap between memantine‐induced transcriptional changes and those in *eat‐2* mutants highlights conserved pathways for potential therapeutic targeting. By uncovering these overlaps in *C. elegans* , our work bridges existing mammalian transcriptomic studies with a systems‐level, whole‐organism view of aging, thus expanding the translational context of memantine's actions. Although memantine has been used for decades in the treatment of Alzheimer's disease, our results suggest its benefits may extend beyond neurodegeneration. Future studies should investigate memantine's translational anti‐aging potential in mammalian models, with particular attention to its effects on metabolic health, stress resilience, and other age‐related pathologies.

## Author Contributions

Vaida Juozaityte and Christoffer Clemmensen conceptualized the project. Chiara Pregnolato, Vaida Juozaityte, and Rebecca L. McIntyre contributed to experimental design, data collection and provided experimental support. RNA‐sequencing studies: Vaida Juozaityte, Steffen Abay‐Nørgaard, Anna Elisabetta Salcini, Dylan Matthew Rausch, Tune H. Pers, Christoffer Clemmensen. All authors contributed to data analysis and interpretation. Vaida Juozaityte and Christoffer Clemmensen wrote the manuscript. All authors provided comments on the manuscript.

## Funding

This work was supported by the Lundbeck Foundation (Fellowship R303‐2018‐3064). Christoffer Clemmensen is supported by a research grant from the Novo Nordisk Foundation, Denmark (Grant NNF22OC0073778). The Novo Nordisk Foundation Center for Basic Metabolic Research is an independent Research Center, based at the University of Copenhagen, Denmark, and partially funded by an unconditional donation from the Novo Nordisk Foundation (http://www.cbmr.ku.dk) (Grant NNF18CC0034900 and NNF23SA0084103).

## Conflicts of Interest

Christoffer Clemmensen is co‐founder of Ousia Pharma, a biotech company developing therapeutics for obesity. The remaining authors declare no competing interests.

## Supporting information


**Figure S1:** Pilot lifespan characterization after memantine exposure. (a) Pilot lifespan assay. Adult worms (L4 + 1 day) were treated with memantine (0.1 mg/mL), memantine (0.25 mg/mL) or control (saline). Treatment with memantine results in median lifespan extension (*n* = 107/control group, *n* = 117/mem 0.1 mg/mL group, *n* = 118/mem 0.25 mg/mL group). Survival analysis was conducted using the Kaplan–Meier method, and statistical significance was analyzed by a log‐rank (Mantel‐Cox) test.
**Figure S2:** Assessment of learning, short‐term and long‐term memory after memantine treatment. (a) Setup of massed learning assay. Well‐fed synchronized worms were collected and starved for 1 h in M9 buffer. After starvation, they were conditioned with food and 10% butanone for 1 h. Worms were then tested for learning immediately after conditioning or were left on hold for different periods to test short‐ and long‐term memory. (b) Learning, *n* = 900/experimental group (c) Short‐term (1 h) memory *n* = 300/experimental group, (d) Long‐term (20 h) memory *n* = 600/experimental group. Results are shown as mean ± SEM and analyzed with ordinary two‐way ANOVA followed by Tukey's multiple comparisons test.

## Data Availability

The RNA‐seq data supporting the findings of this study are openly available at https://github.com/perslab/Juozaityte_2025. All remaining datasets generated and analyzed during the current work are available from the corresponding author upon reasonable request.
