# Supplementary material for: The NMDA Receptor Antagonist Memantine Modulates Aging and Stress Resilience
Source: Aging Cell. 2025 Nov 28;25(1):e70303. doi: 10.1111/acel.70303 (PMC12740082; doi:10.1111/acel.70303)
Supplement: Supplementary file 1 — Figure S1: Pilot lifespan characterization after memantine exposure. (a) Pilot lifespan assay. Adult worms (L4 + 1 day) were treated with memantine (0.1 mg/mL), memantine (0.25 mg/mL) or control (saline). Treatment with memantine results in median lifespan extension (n = 107/control group, n = 117/mem 0.1 mg/mL group, n = 118/mem 0.25 mg/mL group). Survival analysis was conducted using the Kaplan–Meier method, and statistical significance was analyzed by a log‐rank (Mantel‐Cox) test. Figure S2: Assessment of learning, short‐term and long‐term memory after memantine treatment. (a) Setup of massed learning assay. Well‐fed synchronized worms were collected and starved for 1 h in M9 buffer. After starvation, they were conditioned with food and 10% butanone for 1 h. Worms were then tested for learning immediately after conditioning or were left on hold for different periods to test short‐ and long‐term memory. (b) Learning, n = 900/experimental group (c) Short‐term (1 h) memory n = 300/experimental group, (d) Long‐term (20 h) memory n = 600/experimental group. Results are shown as mean ± SEM and analyzed with ordinary two‐way ANOVA followed by Tukey's multiple comparisons test. [file ACEL-25-e70303-s001.docx]

**
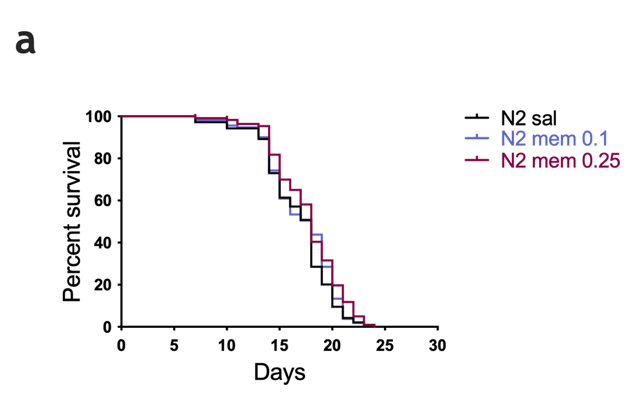
**

**Figure S1. Pilot lifespan characterization after memantine exposure. a,** Pilot lifespan assay. Adult worms (L4 +1 day) were treated with memantine (0.1mg/ml), memantine (0.25mg/ml) or control (saline). Treatment with memantine results in median lifespan extension (n=107/control group, n=117/mem0.1 group, n=118/mem0.25 group). Survival analysis was conducted using the Kaplan-Meier method, and statistical significance was analyzed by a log-rank (Mantel-Cox) test.


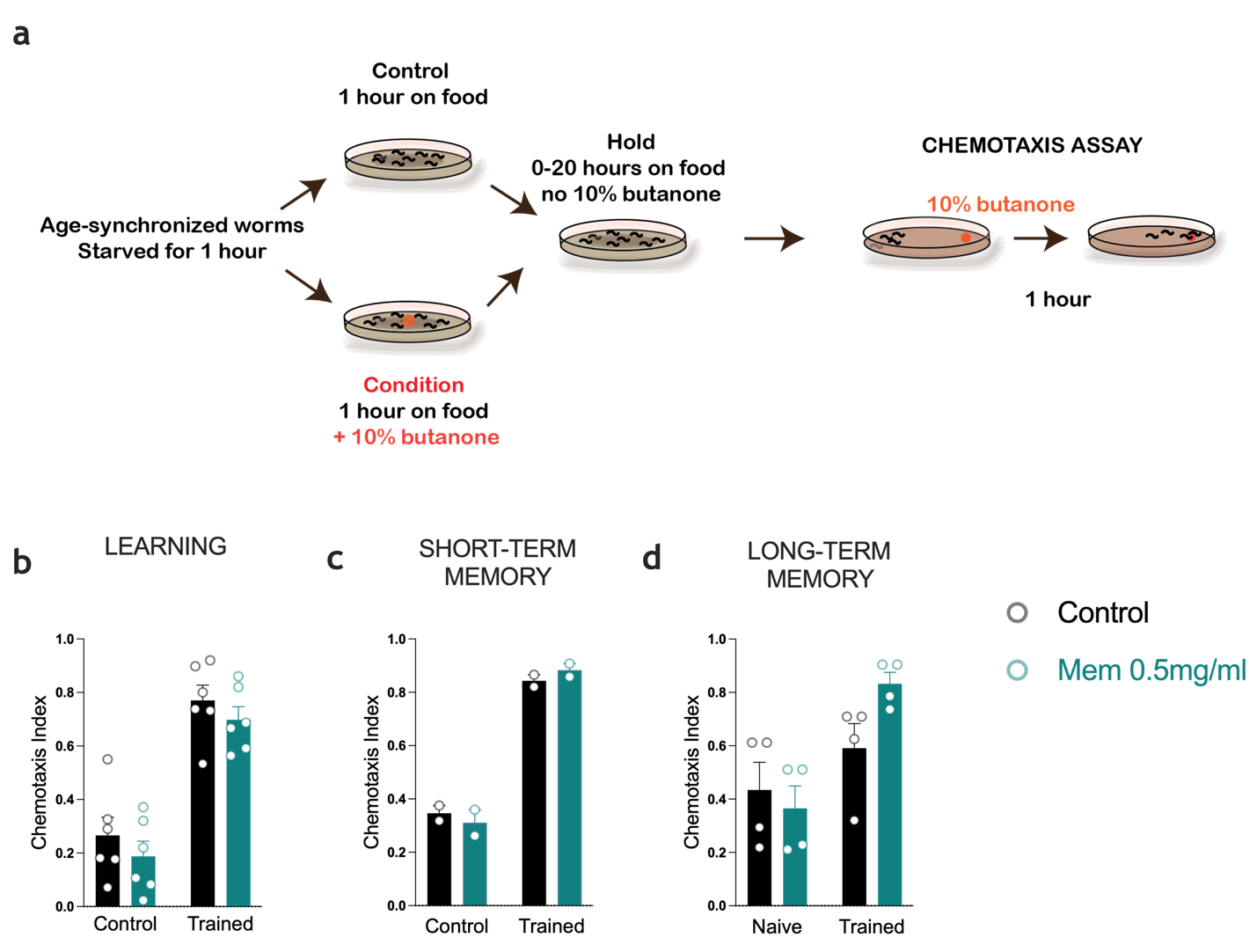


**Figure S2. Assessment of learning, short-term and long-term memory after memantine treatment. a,** Set up of massed learning assay. Well-fed synchronized worms were collected and starved for 1 hour in M9 buffer. After starvation, they were conditioned with food and 10% butanone for 1 hour. Worms were then tested for learning immediately after conditioning or were left on hold for different periods to test short- and long-term memory. **b**, learning, n=900/experimental group **c**, short-term (1h) memory n=300/experimental group, **d**, long-term (20h) memory n=600/experimental group. Results are shown as mean ± S.E.M. and analyzed with ordinary Two-way ANOVA followed by Tukey’s multiple comparisons test.

**Methods**

# ***C. elegans* Strains**

All strains were maintained at 20 °C. The N2 Bristol strain was used as a wild-type strain. Worms were grown on nematode growth medium (NGM). NGM plates were seeded with the E. coli strain OP50 as a food source according to standard procedures and methods (17, 18).

The following *C. elegans* strains were also used: VM487 nmr-1(ak4)II, VC2623 nmr-2(ok3324)V, LIU1 ldrIs1 [dhs-3p::dhs-3::GFP + unc-76(+)], WBM170 wbmEx57 [acs-2p::GFP + rol-6(su1006)], CL2166 dvIs19 [(pAF15)gst-4p::GFP::NLS]III, SJ4100 zcsIs13 [hsp-6::GFP] V, SJ4005 zcIs4 [hsp-4::GFP] V and were obtained from the Caenorhabditis Genetics Center (CGC, University of Minnesota, MN, USA).

**Lifespan assays**

For lifespan assays, 80-100 age-synchronized L4 larvae per condition were placed to fresh NGM agar plates. Day 0 of the lifespan experiment refers to the first day of drug treatment (L4+1) with 400ul of control (saline), memantine (0.5mg/ml) or ketamine (0.5mg/ml). During the first seven days, animals were transferred on new plates containing the same drug concentrations every day to separate from F1s and prevent starvation. After entering the post-reproductive phase, worms were only transferred when necessary. Survival was examined daily by checking avoidance behavior in response to mechanical stimuli or pharyngeal pumping.

## **Learning and memory assays**

Animals of the N2 (Bristol strain, WT) strain, at different life stages (L4 and Day 4 of adulthood) were treated with 400 μL saline or memantine 0.5 mg/mL and kept, with ad libitum access to food, in the incubator at 20°C overnight.

After overnight treatment on plates with ad libitum access to food, animals were collected and starved in M9 buffer (3 g KH_2_PO_4_, 6 g Na_2_HPO_4_, 5 g NaCl, 1 mL 1 M MgSO_4_, H_2_O to 1 liter. Sterilized by autoclaving) for 1 hour. Animals were washed 3 times in the first 10 minutes, and then once every 20 minutes to make sure no food was left in the buffer. After the starving period, animals were placed on 35 mm NGM plates seeded with OP50 bacteria. 1 μL of 2-butanone (Sigma Aldrich) 10% (1: 10 butanone: ethanol 96%) was placed on the lid of the plates for training the animals, while for the control plates, no butanone was added to the lid. Parafilm was placed around the plates and animals were left to crawl for one hour after which the lid was changed to a new clean one. At this point, animals were either tested for chemotaxis to butanone straight after the training for investigating massed learning, or they were left on hold for 1 hour, to test short-term associative memory, and for 24 hours to test long-term memory.

## **Stress Assays**

We evaluated oxidative, mitochondrial and ER stress using fluorescent reporters *gst-4*::GFP, *hsp-6*::GFP and *hsp-4*::GFP, respectively. Nematodes at L4 developmental stage were treated with 400 μL saline or memantine 0.5 mg/mL and were allowed to grow, with ad libitum access to food, in the 20°C incubator overnight. The nematodes were imaged shortly after by light microscope using Zeiss Elyra PS.1 Super Resolution Microscope on 10X magnification with bright field and GFP-channel (488nm).

## **Mobility assay**

Young adults (day 1) were treated overnight with 400 μL saline or memantine 0.5 mg/mL. Afterwards, approximately 30-50 worms were transferred to two NGM plates without OP50, and recorded using an Olympus SZ51 KL 300 LED microscope and AmScope MU130 camera. Recordings were captured using AmLite Software macOS2.1. and then processed with the **Worm Tracker 2.0 system** developed by the Schafer Lab (MRC Laboratory of Molecular Biology, Cambridge, UK). Young adult C. elegans were placed on unseeded NGM plates and allowed to acclimate for 2 minutes before recording. Videos were captured for 20-30sec, and worm speed was analyzed using the accompanying Worm Tracker software. Three different cohorts were used for each experiment.

## **Imaging of reporters using fluorescent microscopy**

Nematodes at L4+1 developmental stage were treated with 400 μL control (saline) or memantine 0.5 mg/mL and were allowed to grow, with ad libitum access to food, in the 20°C incubator overnight. On the day after the treatment animals were imaged using Zeiss Elyra PS.1 Super Resolution Microscope on 10X magnification with bright field and GFP-channel (488nm). At least 40 nematodes were analyzed per cohort in three separate cohorts. Light intensity and time of exposure were kept the same for each cohort. The images were analyzed using Fiji 2.9.0, an open-source platform for biological-image analysis.

## **Statistical Analyses**

The data was collected over separate cohorts and all experiments were repeated at least three times. The results were analyzed by using an unpaired two-tail t-test, one-way ANOVA, or two-way ANOVA with different *post hoc* tests.

Survival analysis with Log-rank test was also executed. P<0.05 was set as a criterion for significance. All data are expressed as mean ± SEM. Analyses were performed using GraphPad Prism version 9.5.1 for MacOS, GraphPad Software, San Diego, California USA.

**Food Race Assay**

After 24 hours of treatment with 400ul of memantine (0.5 mg/ml) or control (saline), age-synchronized adult worms (L4 +2 days) were placed on NMG plates, positioned 5 cm away from a 50 µl OP50 food source. The plates were seeded with food 3 hours prior to the experiment. Approximately 50 worms per treatment group were placed 5 cm away from the food, and every 10 minutes for one hour, the number of worms reaching the food was counted and removed from the plate.

**RNA Sequencing and Analysis**

Immediately after treatment, total RNA was extracted from age-synchronized adult worms (L4 +2 days) using the RNeasy Micro Kit (Qiagen), following the manufacturer’s instructions. Sequencing libraries were prepared using the TruSeq RNA Library Prep Kit v2 (RS-122-2001/2; Illumina), and libraries were sequenced on a NextSeq 500 system using the NextSeq 500/550 High Output Kit v2 (Illumina).

Raw sequencing reads were processed on the Galaxy platform. Adapter trimming and low-quality read removal were carried out using Trim Galore! (v0.6.4) with default settings. Cleaned reads were then aligned to the *C. elegans* reference genome (WBcel235/ce10) using HISAT2 (v2.1.0). Gene-level quantification was performed using featureCounts (v1.6.4) from the Subread package with Ensembl gene annotations (release 98). Only uniquely mapped reads were retained for counting.

Downstream analysis was performed in R using DESeq2 (v1.44.0), with models incorporating batch terms where appropriate. Genes with more than 10 counts in at least 3 samples were retained for downstream analysis. Principal component analysis (PCA) was conducted on the 1000 most variable genes after normalization and batch correction to assess sample clustering by treatment group. To identify genes regulated by either memantine or ketamine, three pairwise comparisons were tested: memantine vs. control, ketamine vs. memantine, and ketamine vs. control. Genes with an absolute log₂ fold change greater than 1.5 and an adjusted p-value below 0.05 were considered significantly differentially expressed. Gene set enrichment analysis was performed using the clusterProfiler(v4.12.6) and msigdbr(v7.5.1) packages in R. Gene sets were obtained from the C5 category of the MSigDB database for *C. elegans*. Over-representation analysis was performed using the compareCluster function to evaluate enriched gene sets across contrasts.

To further characterize expression patterns induced by treatment, we extracted all genes significantly altered by treatment using a likelihood ratio test (LRT) in DESeq2, with a false discovery rate threshold of FDR < 1×10⁻⁵. Variance-stabilized expression values for these genes were scaled and used to generate a heatmap using the ComplexHeatmap (v2.20.0) package in R. Genes were grouped into four expression clusters by k-means clustering. To test the relevance of these pathways, we tested the enrichment of curated gene sets related to pro-longevity, anti-longevity, and feeding behavior (e.g., EAT pathway genes).
